# Spontaneous orientation polarization of flavonoids

**DOI:** 10.1038/s41598-023-46834-1

**Published:** 2023-11-08

**Authors:** Kouki Akaike, Takuya Hosokai, Yutaro Ono, Ryohei Tsuruta, Yoichi Yamada

**Affiliations:** 1 Nanomaterials Research Institute, National Institute of Advanced Industrial Science and Technology, Central 5, Higashi 1-1-1, Tsukuba, 305-8565 Japan; 2 National Metrology Institute of Japan, National Institute of Advanced Industrial Science and Technology, Central 5, Higashi 1-1-1, Tsukuba, 305-8565 Japan; 3 Institute of Pure and Applied Sciences, University of Tsukuba, 1-1-1 Tennodai, Tsukuba, 305-8573 Japan

**Keywords:** Chemical biology, Materials science

## Abstract

Spontaneous orientation polarization (SOP) is macroscopic electric polarization that is attributed to a constant orientational degree of dipole moments of polar molecules on average. The phenomenon has been found in small molecules like H_2_O at low temperatures and π-conjugated molecules employed in organic light-emitting diodes. In this study, we demonstrate that a thin film of baicalein, a flavonoid compound found in natural products, exhibits SOP and resultant giant surface potential (GSP) exceeding 5500 mV at a film thickness of 100 nm. Vacuum-deposition of baicalein under high vacuum results in smooth and amorphous films, which enables the generation of GSP with a slope of 57 mV/nm in air, a value comparable to the representative of an organic semiconductor showing GSP, tris(8-hydroxyquinoline)aluminum(III) (Alq_3_). We also found the superior photostability of a baicalein film compared to an Alq_3_ film. These findings highlight the potential of baicalein in new applications to organic electronics.

## Introduction

Spontaneous orientation polarization (SOP) is a phenomenon observed in gaseous polar molecules, such as water^1,2^ and N_2_O^3,4^, as well as in conjugated molecules with applications in organic light-emitting diodes (OLEDs)^5–8^. This phenomenon arises in amorphous solid states of polar molecules, where molecular permanent dipoles loosely align along the surface normal, resulting in the formation of a giant surface potential (GSP)^5^. A benchmark example is tris(8-hydroxyquinoline)aluminum(III) (Alq_3_), a widely-used electron transport and green-fluorescence material in OLEDs, which exhibits a GSP of 28 V for a 560-nm-thick film under ultra-high vacuum^5^. Recent advancements in controlling the polarity and magnitude of GSP have been achieved through the introduction of side chains^9^, molecular units with low surface energy^6^, and optimization of intramolecular interactions^10^. These developments have paved the way for unique applications of large polar molecules in enhancing hole injection via "dipolar doping"^11^, self-assembled electrets for vibration electric conversion^8^, and prolonging charge lifetimes in OLEDs^12^.

**Figure 1:**
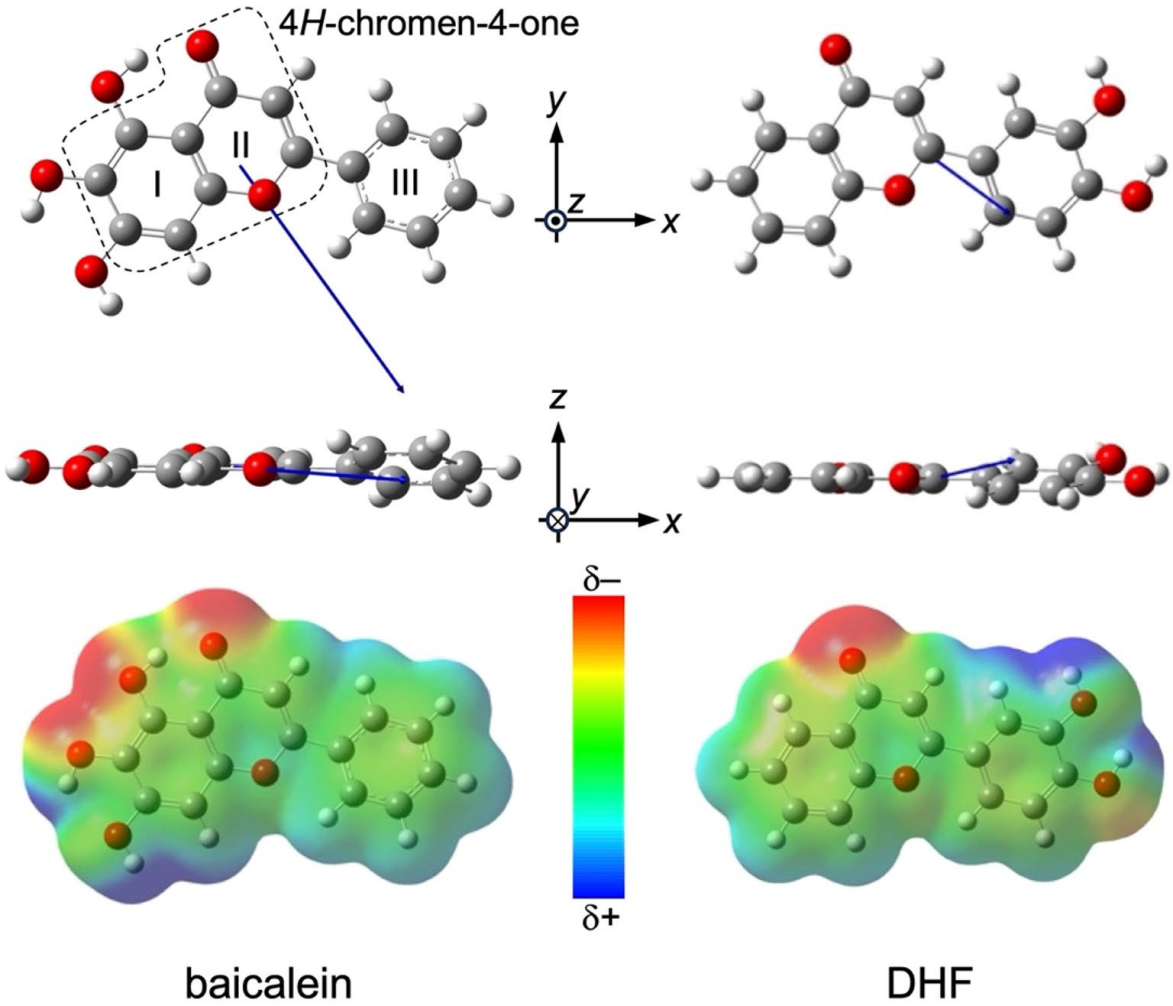
Molecular structures of baicalein and DHF in *xy* (top) and *xz* (middle) planes. White, gray, and red balls denote hydrogen, carbon, and oxygen atoms, respectively. Labels I–III denote three aromatic rings in a baicalein molecule. The fragment encircled by a dashed line indicates 4*H*-chromen-4-one skeleton. Arrows indicate vectors of the permanent dipoles. The dipole moments were calculated by molecular orbital calculations at the B3LYP/6-311G(d,p) level (see Experimental Section). Electron density maps of baicalein and DHF (isovalue = 0.001 e Å^−3^) in *xy* plane are also shown at the bottom for visual assistance to recognize the direction of the permanent dipoles.

Interestingly, nature also harbors a vast array of polar molecules, such as phenylpropanoids and polyphenols. Our recent study demonstrated that a thin layer of caffeic acid (CfA), which has a dipole moment of 4.34 D, could effectively increase electrode work functions and enhance hole injection into *p*-type organic semiconductors from prototypical electrodes^13^. However, unlike Alq_3_, CfA does not exhibit GSP; instead, a dipole layer is formed only near the electrode due to preferential interactions between the catechol moiety of CfA and electrode surfaces^13^. These findings prompted us to seek natural molecules capable of reducing electrode work functions and being applicable to a buffer layer for electron-injection electrodes. We selected baicalein (molecular structure shown in Fig. 1) as a candidate for this purpose. Baicalein is one of the flavonoids, a group of natural substances widely found in fruits and vegetables^14^ and consists of a phenyl group bonded to a 4*H*-chromen-4-one skeleton with three hydroxy groups on the benzene ring. The molecule has a dipole moment of 7.08 D whose vector is parallel to the *xy* plane of 4*H*-chromen-4-one skeleton; the direction is indicated in Fig. 1. Since baicalein forms metal complexes with oxovanadium^15^, iron^16^, aluminum^17^, nickel^18^, and ruthenium^19^ via coordination bonds of 4*H*-chromen-4-one skeleton, the preferential interaction between the skeleton and electrode surfaces expectedly forms a dipole layer with the positive charges on the outermost surface, and thereby reduce the work function of electrodes.

In this study, we indeed demonstrated the lowering of the surface potential upon the deposition of baicalein onto electrodes such as indium-tin oxide (ITO), but, surprisingly, the vacuum-deposited film of baicalein generates GSP with a slope of 57 mV/nm in air. Remarkably, the GSP slope of the baicalein film is comparable to a reported GSP of an Alq_3_ film measured under ultra-high vacuum. The stability of the GSP was investigated in ambient conditions under air mass (AM) 1.5 simulated solar light irradiation at 1 sun. The GSP of a baicalein film remains over 30 min, whereas the GSP of an Alq_3_ film decays less than 10 s. The superior photostability of the baicalein’s GSP paves the way for the application of baicalein to organic electronics.

## Results and discussion

**Figure 2:**
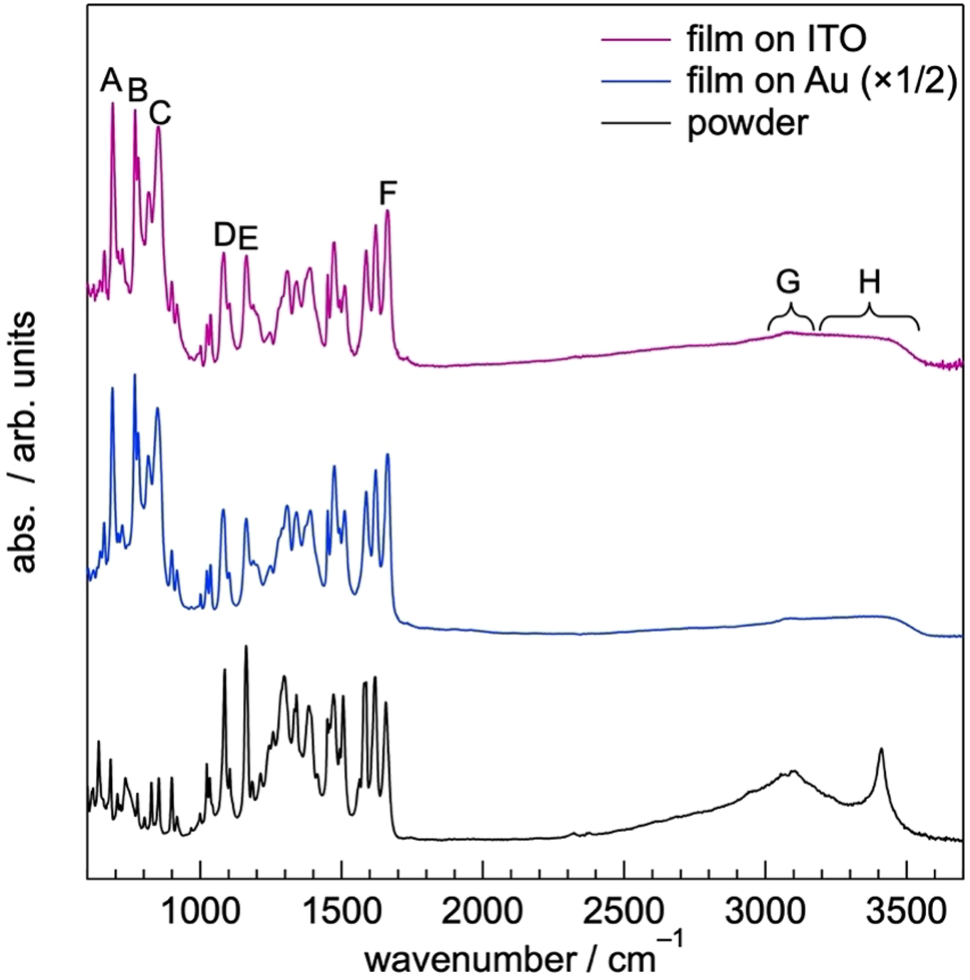
FTIR-RAS spectra of vacuum-deposited baicalein films of 100 nm on ITO and Au substrates. FTIR spectrum of powdery baicalein is also shown for comparison.

At first, we tested vacuum deposition of baicalein under high vacuum (< 5 × 10^−4^ Pa). Figure 2 show Fourier-transform infrared (FTIR) spectra of the evaporated films of 100 nm prepared on ITO and Au substrates. The spectra were acquired by reflection absorption mode (FTIR-RAS). Overall, the spectral feature is similar to the FTIR spectrum of the powdery baicalein (bottom spectrum in Fig. 2). Characteristic vibrational absorptions of baicalein, which are assigned to out-of-plane bending of C–H bonds in the ring III in a baicalein molecule (690 and 769 cm^−1^, the locations of the rings I–III are found in Fig. 1), out-of-plane bending of the C–H bond in the ring II and out-of-plane bending of an O–H bond in the ring I (851 cm^−1^), C–O stretching of ring II, in-plane bending of C-H bonds in the ring III (1082 cm^−1^), and in-plane bending of C–H bonds in the rings I–III (1163 cm^−1^), C=O stretching (1663 cm^−1^), C–H stretching of aromatic rings (3000–3200 cm^−1^), and O–H stretching (3200–3600 cm^−1^), are clearly observed. The assignment of these peaks was done in the reference to a previous literature^20^ and the vibrational analysis of DFT calculation at the B3LYP/6-311G(d,p) level (Table 1). This result suggests that the chemical structure of baicalein in the evaporated film is maintained. Note that the absorbances of aromatic C–H and O–H stretching in the FTIR-RAS spectra of the films are significantly lower than that observed in the FTIR spectrum of the bulk baicalein (Fig. 2). Concurrently, the vibrational absorbance derived from out-of-plane bending of C–H and O–H bonds (peaks A–C) in the film spectra are strongly enhanced in comparison to the powdery sample. Since the IR light used in FTIR-RAS measurements is *p*-polarized, the absorbances of vibrations having the transition dipole moment along to the surface normal increase. Thus, the comparison of the vibrational spectra of the evaporated films and the bulk indicates that the molecular plane of baicalein in the films is nearly parallel to the substrate surfaces.

**Table 1:** Assignment of representative vibrational absorptions labelled as A–H in Fig. 2. The calculated wavenumbers were by using a scaling factor (0.9682)^21^.

Label	Experimental wavenumber/cm^−1^	Calculated wavenumber/cm^−1^	Assignment
A	690	684	C–H out-of-plane bending of ring III
B	769	764	C–H out-of-plane bending of ring III
C	851	842	C–H out-of-plane bending of ring II, O–H out-of-plane bending of ring I
D	1082	1082	C–O stretching of ring II, C–H in-plane bending of ring III
E	1163	1161	C–H in-plane bending of rings I–III
F	1663	1652	C=O stretching
G	3000–3200	3075–3125	aromatic C–H stretching
H	3200–3600	3175–3727	O–H stretching

**Figure 3:**
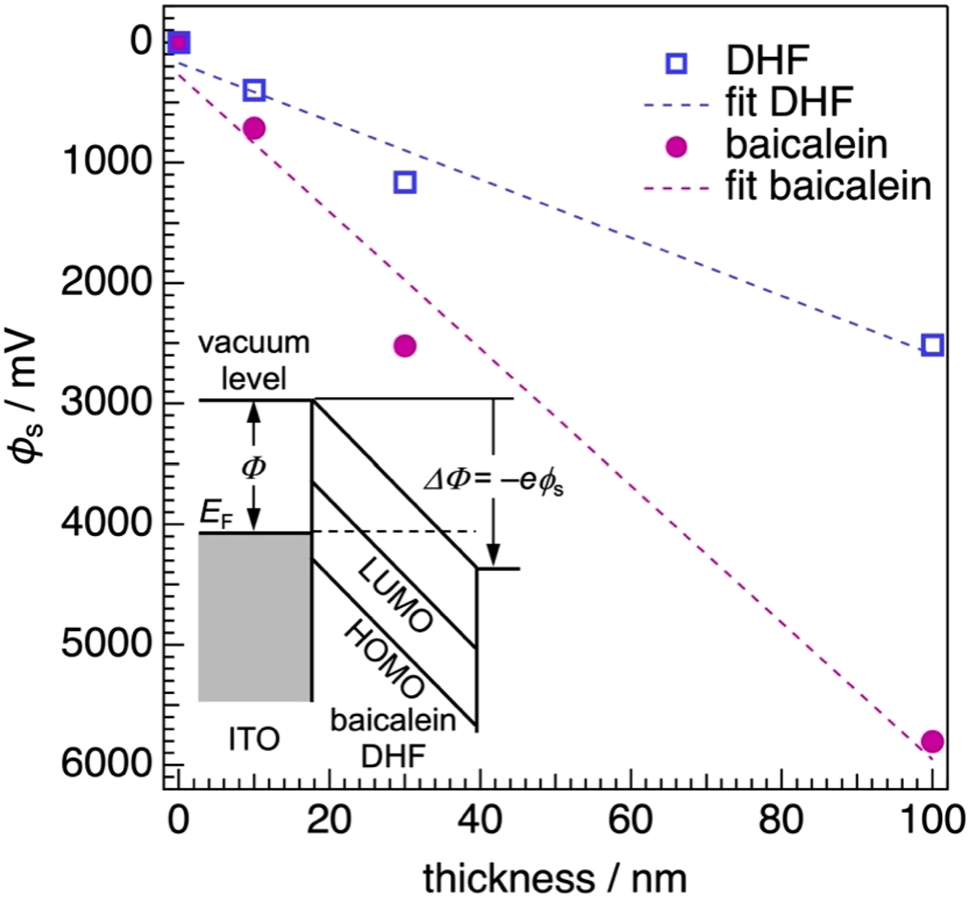
The evolution of surface potential *ϕ*_s_ for ITO substrates as a function of thicknesses of baicalein and DHF. The dashed lines indicate linear fitting of the measured *ϕ*_s_. The inset illustrates the definition of *ϕ*_s_. The sign of *ϕ*_s_ becomes positive when work function *Φ* decreases (*ΔΦ* < 0). *E*_F_ denotes the Fermi level of an ITO substrate. The HOMO and LUMO means the highest occupied molecular orbital and lowest unoccupied molecular orbital, respectively.

The deposition of baicalein onto substrates lowers their electrostatic potential as we expected. Figure 3 shows the evolution of surface potential (*ϕ*_s_) as a function of nominal film thickness of baicalein. Here, *ϕ*_s_ is defined as the change in the electrostatic potential of a clean substrate^5^. When work function (*Φ*) decreases upon the deposition of an overlayer (in other words, *ΔΦ* < 0), the sign of *ϕ*_s_ becomes positive through the relation of *ΔΦ* = –*eϕ*_s_ (see the inset in Fig. 3). In this study, *Φ* was determined by Kelvin probe (KP) under ambient conditions. Upon the deposition of baicalein with the thickness of 10 nm onto a clean ITO substrate, *ϕ*_s_ increases by 410 mV. With increasing the baicalein thickness, unlike the case of CfA^13^, *ϕ*_s_ does not saturate and continue to increase monotonically: the value for 100-nm-thick baicalein film reaches 5803 mV. The linear behavior of *ϕ*_s_ measured upon the deposition of baicalein suggests the emergence of GSP. The build-up of the positive GSP is independent of substrate kinds. 100-nm-thick films of baicalein evaporated on Au film, highly oriented pyrolytic graphite and *n*-doped silicon wafer with a native oxide layer (SiO_x_) produce GSPs of 7272, 4817 and 6885 mV, respectively. The variation of the measured GSPs with the used substrates implies that surface properties, such as roughness and surface energy, may affect SOP.

Since baicalein is one of the flavonoids, we expected that flavone also generated GSP upon film formation. However, a flavone film was not formed on an ITO substrate at room temperature even when increasing the evaporation rate by ten times the baicalein case, 10 Å/s, to suppress the desorption of flavone molecules from the substrate. 3’,4’-dihydroxyflavone (DHF, molecular structure shown in Fig. 1), instead, formed an evaporated film stable at room temperature. The improvement of the film stability should be attributed to the increase in intermolecular interaction through hydrogen bonds via catechol groups of DHF. We found that DHF also generated GSP but the positive shift of *ϕ*_s_ was moderate (Fig. 3); *ϕ*_s_ for 100-nm-thick DHF film on an ITO substrate is 2513 mV, which is the half of the GSP for the baicalein film with the same thickness.

Analysis of the slope of thickness (*d*)-*ϕ*_s_ relation can deduce information on orientational order in the baicalein film. *ϕ*_s_ is described by the following Eq. ^7^,

1 $${\phi }_{\mathrm{s}}=\frac{n p \langle \mathrm{cos}\omega \rangle d}{\epsilon }$$

where *n*, *p*, < cos*ω* > , *d*, and *ε* denote molecular density, permanent dipole, first-order orientational parameter with the angle with respect to the surface normal, film thickness, and dielectric constant. A slope of GSP of the baicalein films on an ITO substrate was calculated by a linear fitting (dashed line in Fig. 3) and found to be 57 mV/nm, which is comparable to the reported value of Alq_3_ measured in ultra-high vacuum (33–50 mV/nm)^5,9,22^. For DHF, the GSP slope is 24 mV/nm. Since the permanent dipole moment of DHF (2.62 D) is smaller than baicalein (7.08 D), the slope value decreases according to Eq. (1). Assuming the density of baicalin in single crystals of form α^23^ and typical relative permittivity of 3.5 for typical organic semiconductors^24^, < cos*ω* > is found to be 0.022. This value is less than half smaller than that of Alq_3_ (0.05). The small < cos*ω* > ruins the positive impact of the large permanent dipole of a baicalein molecule and then decreases the GSP slope.

**Figure 4:**
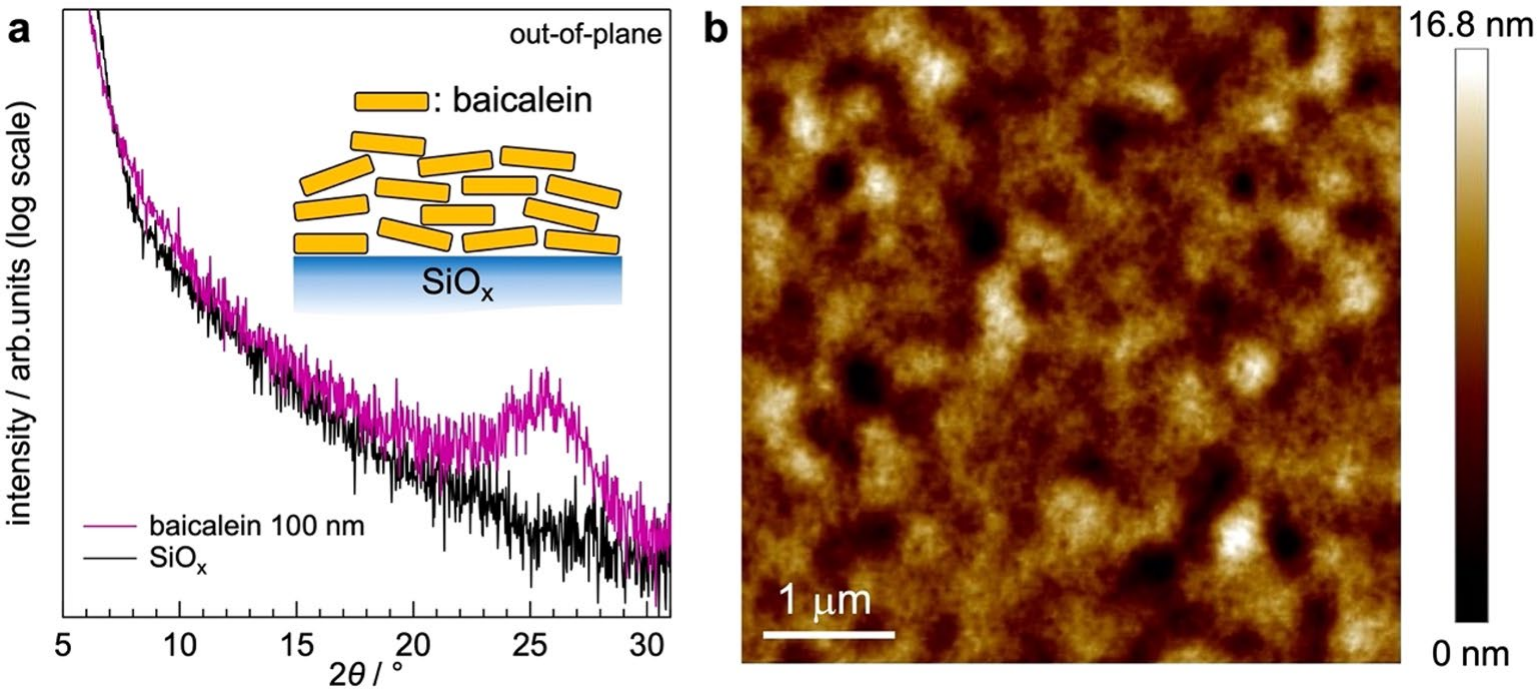
(**a**) XRD profiles of baicalein evaporated film of 100 nm and bare SiO_x_ substrate. Inset schematically illustrates molecular arrangement of baicalein in the evaporated film. (**b**) AFM image of 60-nm-thick baicalein film prepared on an ITO substrate.

To gain insights of molecular orientation that explains the small < cos*ω* > , we measured out-of-plane X-ray diffraction (XRD) of 100-nm-thick baicalein film evaporated on SiO_x_ (Fig. 4a). Only a weak and broad diffraction was found at 25.73º in logarithmic scale; The value is close to the two diffractions reported for a single crystal of baicalein in form α at 23.9 and 26.4°^23^. The large full-width-at-half-maximum of the observed peak means that the film is likely amorphous. The surface morphology of the baicalein film was investigated by atomic force microscopy (AFM) and found to be smooth (Fig. 4b). Root-mean-square roughness of the film surface is 2.28 nm, which supports the amorphous nature. A short-range order, however, exists in the evaporated film of baicalein. *d*-spacing calculated for the observed peak is 3.46 Å, which is in the range of π-π stacking. This result suggests that the molecular plane of baicalein is nearly parallel to the substrate surface (inset in Fig. 4a). The observed orientation agrees with the FTIR-RAS spectra (Fig. 2), where the vibrational absorption of the stretching modes of aromatic C–H and O–H bonds is quite low in contrast to the FTIR spectra of bulk. Since hydrogen bonding via hydroxyl and carbonyl groups exists between baicalein molecules neighboring in the direction lateral to the molecular plane^23^, 2D molecular layers stabilized by the intermolecular interaction are stacked on substrates. As a result, baicalein molecules adopt a face-on orientation. An ideally face-on orientation, however, does not produce GSP because the permanent dipole of baicalein almost parallel to the molecular plane (Fig. 1) hardly contributes to the SOP along the surface normal. We thus infer that baicalein molecules are slightly inclined from the substrate surface plane (inset in Fig. 4a) and that the imperfect orientation of the permanent dipoles produces the GSP. The nearly face-on orientation gives *ω* close to 90°. Besides, the amorphous nature of baicalein evaporated films should have a broad distribution of intermolecular distance along the surface normal, which varies cos*ω*. Therefore, the expectedly large and various *ω* leads to the small < cos*ω* > .

**Figure 5:**
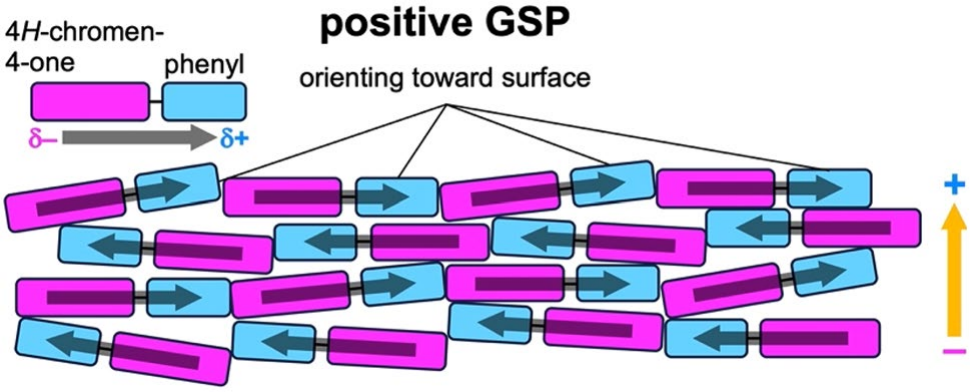
Schematic illustration of molecular orientation in an evaporated film of baicalein.

We were also intrigued by the fact that the signs of the GSPs for baicalein films were always positive, independent of substrate types. In principle, baicalein molecules inclined to the substrate surface may also lead to the negative GSP, that is, the raise of *Φ*, if *ω* exceeds 90°. The KP results shown in this study are, however, not the case. The positive GSPs of baicalein can be related to the molecular structure of baicalein (Fig. 1): In reference to a previous study^6^, we infer that non-polar phenyl group should exhibit lower surface energy in comparison to the polar 4*H*-chromen-4-one skeleton. A large fraction of baicalein molecules thus preferentially exposes the former groups toward the outermost surface, which forces the molecular planes to incline from the substrate surface with the dipole vectors pointing slightly upward (Fig. 5). Such molecular orientation almost cancels out the horizontal components of permanent dipole vectors, while those normal to the substrate add up as film thickness increases. The latter contribution consequently leads to the positive GSP.

In the case of DHF, however, reasoning the positive GSP is difficult because the difference in surface energy between the catechol and 4*H*-chromen-4-one skeleton is not concisely recognized. Further investigation is necessary to elucidate the positive GSP observed for the evaporated film of DHF.

**Figure 6:**
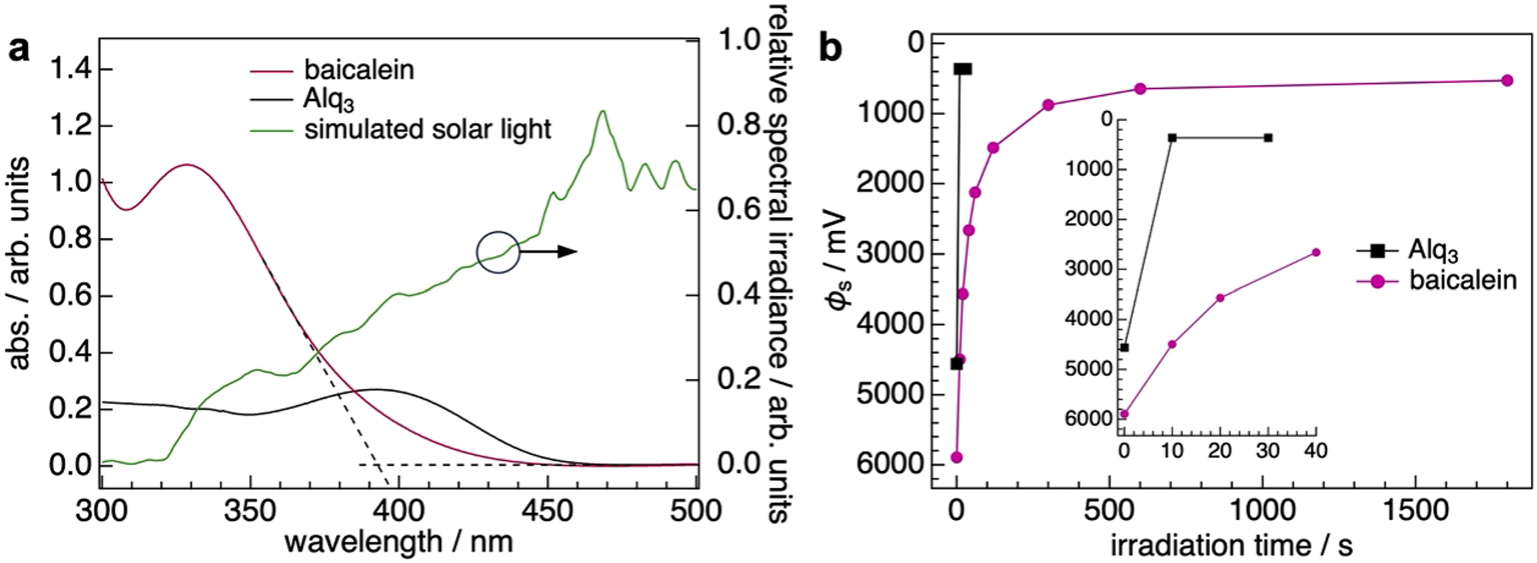
(**a**) UV–Vis spectra of evaporated films of baicalein and Alq_3_ prepared on quartz substrates. Thicknesses of both films are 100 nm. Onsets of optical absorptions were determined by the cross points of tangents and baselines indicated by dashed lines representatively for the spectrum of the baicalein film. Relative spectral irradiance of the simulated solar light is also plotted. The contribution of the photons below 320 nm is negligible. (**b**) Photostability tests of GSP for the baicalein and Alq_3_ films of 100 nm under the illumination of simulated solar light at 1 sun in air. The inset shows the evolution of *ϕ*_s_ within 40 s.

Finally, we investigated the photostability of the GSP for the baicalein film because the potential is known to disappear when polar molecules absorb visible light and generate photo-induced charges that compensate polarization charges^5^. Ultraviolet–visible (UV–Vis) absorption spectroscopy clarified that a baicalein film had a wider optical gap (*E*_g,opt_) than Alq_3_ film (Fig. 6a). *E*_g,opt_ of a baicalein was calculated to be 3.2 eV, which was determined from the spectral onset at the longer wavelength. The obtained value is 0.5 eV larger than that of Alq_3_, which indicates that the positive GSP of the baicalein film is more tolerant to that of the Alq_3_ film under visible-light illumination because the amount of the photogenerated compensation charges could be less than the Alq_3_ case. Figure 6b shows the time dependence of *ϕ*_s_ for the baicalein and Alq_3_ films of 100 nm under the illumination of the simulated solar light at 100 mW cm^−2^ in air. As we expected, the GSP of the baicalein film remains much longer than that of the Alq_3_ film. GSP of the Alq_3_ film completely disappears within only 10 s (the inset in Fig. 6b), while *ϕ*_s_ of 2123 mV was measured for the baicalein film even at 60 s and the GSP disappeared within 1800s. The result clearly indicates the superior photostability of the positive GSP of the baicalein film to that of the Alq_3_ film.

Unlike TPBi case^8^, the spectral irradiance of the light used in this study overlaps with the UV–Vis spectra of baicalein and Alq_3_ films (Fig. 6a); The photons from 320 to 470 nm generate the photo-induced compensation charges. The absorbances of the two films in this wavelength range are largely different, although the film thicknesses are identical. This fact complicates the reasoning that the wider *E*_g,opt_ of the baicalein film accounts for the superior photostability of the positive GSP of the baicalein film. To substantiate the result, it is necessary to characterize the generation of the compensation charges in baicalein and Alq_3_ films under illumination. First, absorption efficiency *x*_abs_(*λ*), defined as 1–10^−*A*(*λ*)^, where *A*(*λ*) is an absorbance at a certain wavelength, *λ*, was calculated from the UV–Vis spectra shown in Fig. 6a (see Supplementary Information). *x*_abs_(*λ*) is then multiplied by the relative spectral irradiance of the simulated solar light used in this study (Fig. 6a). The calculated product, denoted as *N*(*λ*), corresponds to the number of excitons generated upon photo-absorption in the films under the assumption that exciton generation efficiency is 100%. Given the same efficiency of the charge separation in the baicalein and Alq_3_ films under the comparable electric field of the positive GSPs, *N*(*λ*) roughly estimates the amount of the photo-induced charges.

Figure S1 in Supplementary Information shows the result. *N*(*λ*) peaks at the absorption maxima of baicalein and Alq_3_ in 300–470 nm. The integral over this range corresponds to the sum of the photo-induced charges generated. The calculations showed that the integral of *N*(*λ*) was ~ 14 for both films, indicating the generation of a similar amount of compensation charges in the baicalein and Alq_3_ films. Therefore, other factors lead to the difference in the photostability of the GSPs. One of the possibilities is the faster bleaching of the Alq_3_ film under the intense illumination by a solar simulator. This possibility is, however, ruled out because the change in the UV–Vis spectra of the film is negligible even when the simulated solar light irradiates the Alq_3_ film for 120 s [Figure S2(a), Supplementary Information]; That time is much longer than the photo-induced decay of the GSP of Alq_3_.

**Figure 7:**
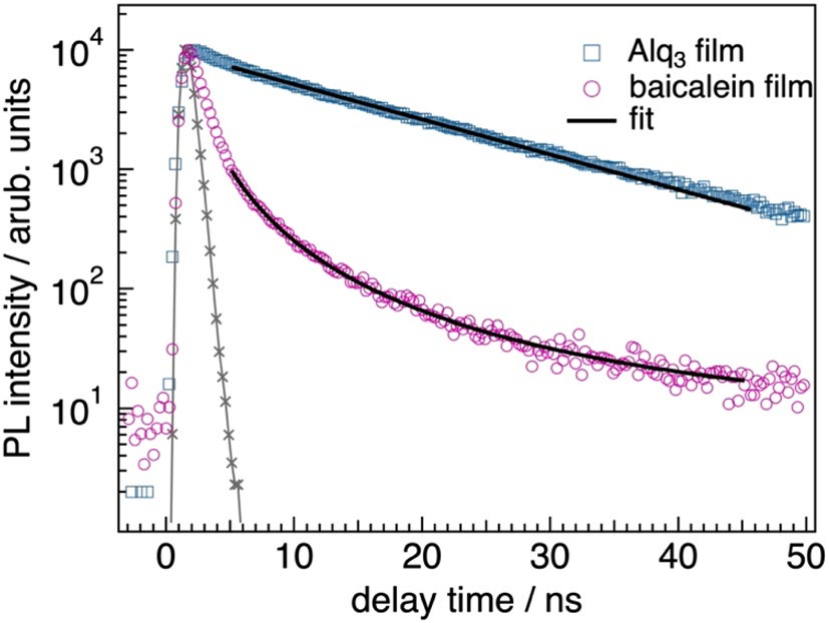
The PL decay profiles of Alq_3_ and baicalein films of 100 nm. The films were excited at 342 nm and PLs at 460 and 500 nm were detected for the baicalein and Alq_3_ films, respectively. The steady-state PL spectra are shown in Fig. S3 of Supplementary Information. The gray curve shows an instrumental response function. The decays of the Alq_3_ and baicalein films were fitted with an exponential and power law, respectively.

We also suppose that actual numbers of generated excitons are deviated from the estimated *N*(*λ*) due to the difference in exciton lifetimes between the baicalein and Alq_3_ films. We thus carried out time-resolved photoluminescence (TR-PL) measurements for 100-nm-thick films of baicalein and Alq_3_ (Fig. 7). The steady-state PL spectra are shown in Fig. S3 of Supplementary Information. It is evident from Fig. 7 that the PL of the baicalein film at 460 nm decays faster than that of the Alq_3_ film at 500 nm. This indicates that lifetimes of excitons (*τ*_ex_) generated in the baicalein film are shorter compared to Alq_3_. To extract *τ*_ex_, the measured profile (*I*(*t*)) of the Alq_3_ film was fitted with an exponential decay, *I*(*t*) = *A* exp (–*t*/*τ*_ex_), where *A* is the fraction of the excited state. The fitting analysis found that *τ*_ex_ for the Alq_3_ film was 15 ns, whose value well agrees with a previous report^25^. On the other hand, the PL decay of the baicalein film does not obey an exponential but a power law. Origins of a power-law PL decay vary widely, such as relative variance of lifetime fluctuation^26^ and exponential distribution of barrier heights for electron injection at interfaces of dissimilar materials^27^, detailing the origins of the power-law decay is beyond the scope of this study. We thus focus on the shorter lifetime of excitons generated in the baicalein film, as evidenced by the steep PL decay observed for the baicalein film, which should be related to the superior photostability of the baicalein’s GSP. The TR-PL results can be interpreted by an efficient free-charge separation or deactivation of excitons. The former possibility is unlikely because typical dielectric constant organic molecules are commonly 3–5, which does not make significant differences in an exciton binding energy. The latter case can thus account for the faster PL decay although fates of excitons generated in the baicalein film are elusive at present. Based on the abovementioned results and discussion, the better photostability of the positive GSP for the baicalein film is attributed to the smaller number of the compensation charges because of the shorter lifetime of the excitons than Alq_3_.

In conclusion, our study has demonstrated the generation of the positive GSP by a natural polar molecule, baicalein, beyond our expectation that the adsorption of the molecules onto the electrodes reduces work function due to the preferential interaction of 4*H*-chromen-4-one skeleton with the electrode surfaces. The sublimation of baicalein under high vacuum results in smooth and amorphous films with the GSP slope of 57 mV/nm. The molecules incline to the substrate surface in the evaporated film, in which the non-polar phenyl group of baicalein can orient toward the surface due to its lower surface energy than the polar 4*H*-chromen-4-one skeleton. Although *ω* is expectedly large and orientational distribution is broad due to the amorphous nature of the evaporated film, with the large permanent dipole exceeding 7 D, the small < cos*ω* > can be utilized to exhibit the positive GSP with the magnitude comparable to Alq_3_. Our finding that even polar molecules that plants biosynthesize can exhibit GSP expands the scope of SOP studies to natural products. Besides, the GSP of a baicalein film is more stable than that of an Alq_3_ film under the illumination of simulated solar light in the air. TR-PL measurements clarified that excitons generated in a baicalein film deactivate faster than those in an Alq_3_ film. As a result, the actual number of the compensation charges in the baicalein film should be small compared to Alq_3_ case. Such superior photostability will stimulate studies on the applicability of baicalein to, for instance, a cathode buffer layer in an organic solar cell because a positive GSP may cause the downward energy shift of the LUMO (as shown in the energy diagram of Fig. 3) and, thereby, facilitate electron extraction toward cathode electrode^28–30^.

## Methods

### Materials

Flavone and DHF were purchased from Thermo Fisher Scientific Inc. Baicalein and Alq_3_ were purchased from Cayman Chemical, and Sigma-Alrdich., respectively. All the materials were used without further purification. ITO, Au, HOPG, and SiO_x_ substrates were purchased from Photo Precision Corp., Geomatec Co., Ltd., Crystal Base Co., Ltd. and Nilaco Corp., respectively. Prior to use, all substrates except HOPG were ultrasonicated in pure water, acetone, and isopropanol for 15 min. each. Clean surfaces of HOPG were obtained by exfoliation in air. Baicalein and DHF were vacuum deposited at a rate of 1 Å/s on the substrates. For UV–visible absorption spectroscopy, baicalein and Alq_3_ of 100 and 50 nm, respectively, were evaporated at a rate of 1 Å/s onto quartz substrates (SEIREN KST Corp.), respectively. Nominal thickness and evaporation rate were monitored with a quartz-crystal microbalance.

### Characterization

KP measurements were conducted in air with a commercial probe (KP020, KP Technology). The off-null method was used to determine the contact potential difference by the linear interpolation of the output responses at the two backing potentials^31^. WF measurements with KP were carried out for the substrates covered with the polar molecules of various thicknesses. FTIR spectra of bulk baicalein was measured in attenuated total reflection (ATR) mode using a Ge prism in an FTIR spectrometer (FT/IR-6600, JASCO). FTIR-RAS spectra were acquired with the same spectrometer. The angle of incidence was 85º with respect to the surface normal. The reflected light was detected with a mercury–cadmium–telluride (MCT) detector. Surface morphology of a baicalein film was analyzed by AFM (Innova, Veeco) in tapping mode under ambient conditions. UV–visible absorption spectra were collected using a spectrophotometer (V-750, JASCO). XRD analyses for a baicalein film of 100 nm on SiO_x_ was carried out with a diffractometer (SmartLab, Rigaku) using Cu Kα as a radiation source. For the photostability tests, baicalein and Alq_3_ films of 100 nm deposited on clean ITO substrates were illuminated under AM 1.5 simulated solar light irradiation at an intensity of 100 mW cm^−2^ (HAL-320, Asahi Spectra). Steady-state PL spectra were corrected by a commercial fluorescence spectrometer (FP-8550, JASCO). TR-PL measurements (FluoroCube, HORIBA) were performed at room temperature (297 K). A pulsed laser diode (NanoLED, HORIBA; wavelength: 342 nm, pulse width: 1.2 ns, pulse energy: 1–2 pJ, repetition rate: 100 kHz) was used as the excitation light source. The sample films were irradiated with the excitation light passed through a short-pass filter (cut-off wavelength: 360 nm) at 30º relative to the surface normal of the samples. The emitted PL was detected at 60º relative to the surface normal through a long-pass filter (cut-on wavelength: 370 nm).

### Theoretical calculations

Molecular orbital calculations were performed using the GAUSSIAN09 package. DFT calculations of baicalein and DHF molecules were performed using the B3LYP exchange–correlation function and 6–311G(d,p) basis set after structural optimization with the same calculation conditions.

### Supplementary Information

Supplementary Figures.

## Data Availability

All data generated or analyzed during this study are included in this published article.
