# Supplementary material for: Spontaneous orientation polarization of flavonoids
Source: Sci Rep. 2023 Nov 8;13:19402. doi: 10.1038/s41598-023-46834-1 (PMC10632337; doi:10.1038/s41598-023-46834-1)
Supplement: Supplementary file 1 — Supplementary Figures. [file 41598_2023_46834_MOESM1_ESM.docx]

Supporting Information

**Spontaneous orientation polarization of flavonoids**

*Kouki Akaike**, *Takuya Hosokai, Yutaro Ono, Ryohei Tsuruta, and Yoichi Yamada*

K. Akaike

Nanomaterials Research Institute, National Institute of Advanced Industrial Science and Technology, Central 5, Higashi 1-1-1, Tsukuba, 305-8565, Japan
E-mail: kouki.akaike@aist.go.jp

T. Hosokai

National Metrology Institute of Japan, National Institute of Advanced Industrial Science and Technology, Central 5, Higashi 1-1-1, Tsukuba, 305-8565, Japan

Y. Ono, R. Tsuruta, Y. Yamada

Institute of Pure and Applied Sciences, University of Tsukuba, 1-1-1 Tennodai, Tsukuba, 305-8573 Japan

*Corresponding author:

kouki.akaike@aist.go.jp (K.A.)

**Estimation of excitons generated upon photo-absorption**

We first calculated the absorption efficiency, *x*_abs_(*λ*), from the measured UV-Vis spectra. This accounts for how much photons at a wavelength (*λ*) are absorbed by a sample. Therefore, *x*_abs_(*λ*) is related to transmittance *T*(*λ*) by *x*_abs_(*λ*) = 1–*T*(*λ*). Since *T*(*λ*) is defined as *T*(*λ*) = *I*(*λ*)/*I*_0_(*λ*), where *I*_0_(*λ*) and *I*(*λ*) denote intensities of the incident light and light after photo-absorption by a sample, *x*_abs_(*λ*) can be related to absorbance *A*(*λ*) by *x*_abs_(*λ*) = 1–10^–^*^A^*^(^*^λ^*^)^. The calculated *x*_abs_(*λ*) is then multiplied by relative spectral irradiance of the simulated solar light. The resulting product, denoted as *N*(*λ*), can quantify the number of the excitons formed upon photo-absorption under the assumption that the exciton generation efficiency is 100%. *N*(*λ*) of baicalein and Alq_3_ films are plotted in Fig. S1. Given the same efficiency of the charge separation of the excitons under the high electric fields of the GSPs of the Alq_3_ and baicalein films, the integral of *N*(*λ*) from 320 to 470 nm becomes a rough measure of the number of the photo-generated compensation charges.

**
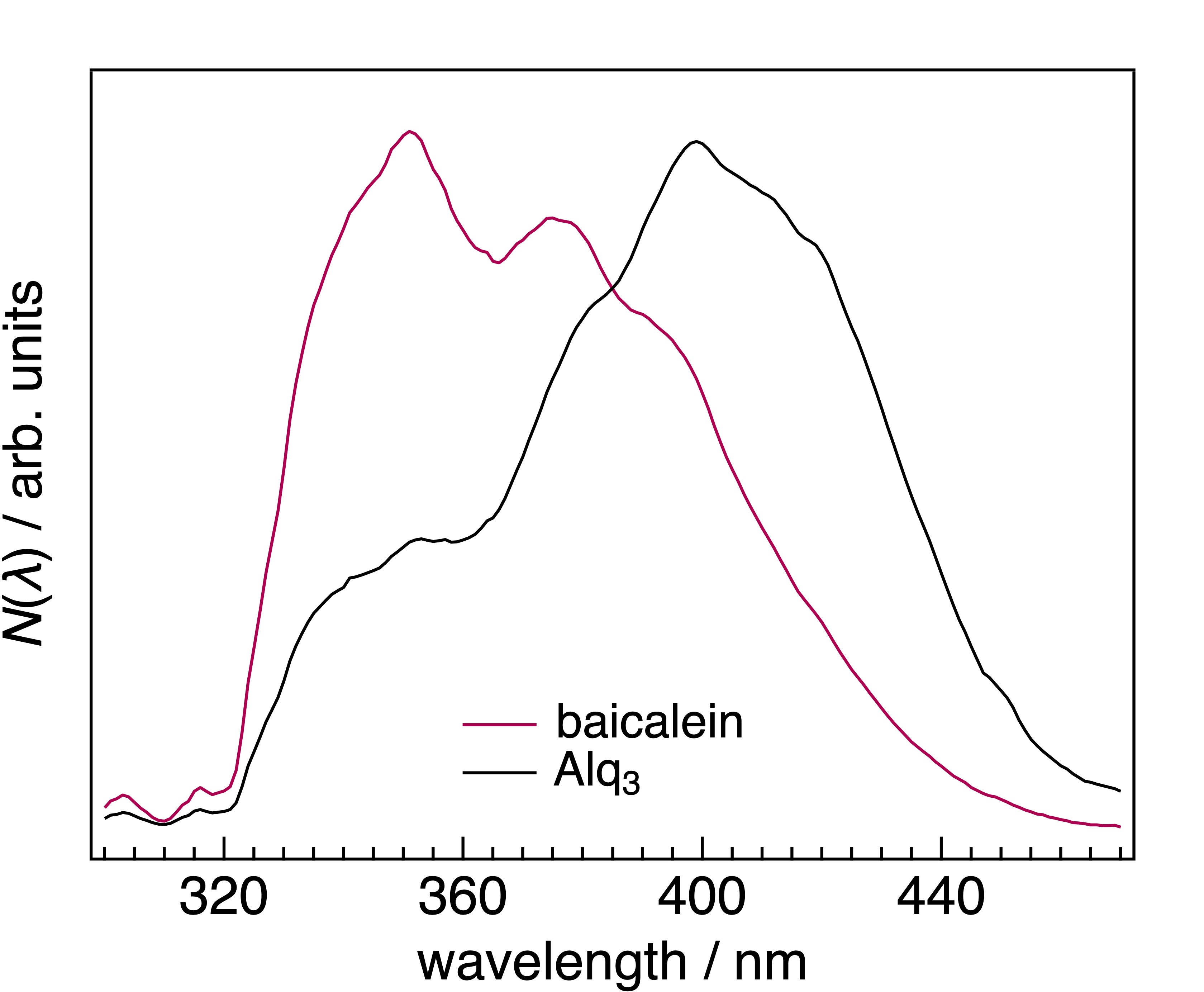
**

**Figure S1**. Comparison of *N*(*λ*) for baicalein and Alq_3_ evaporated films of 100 nm.


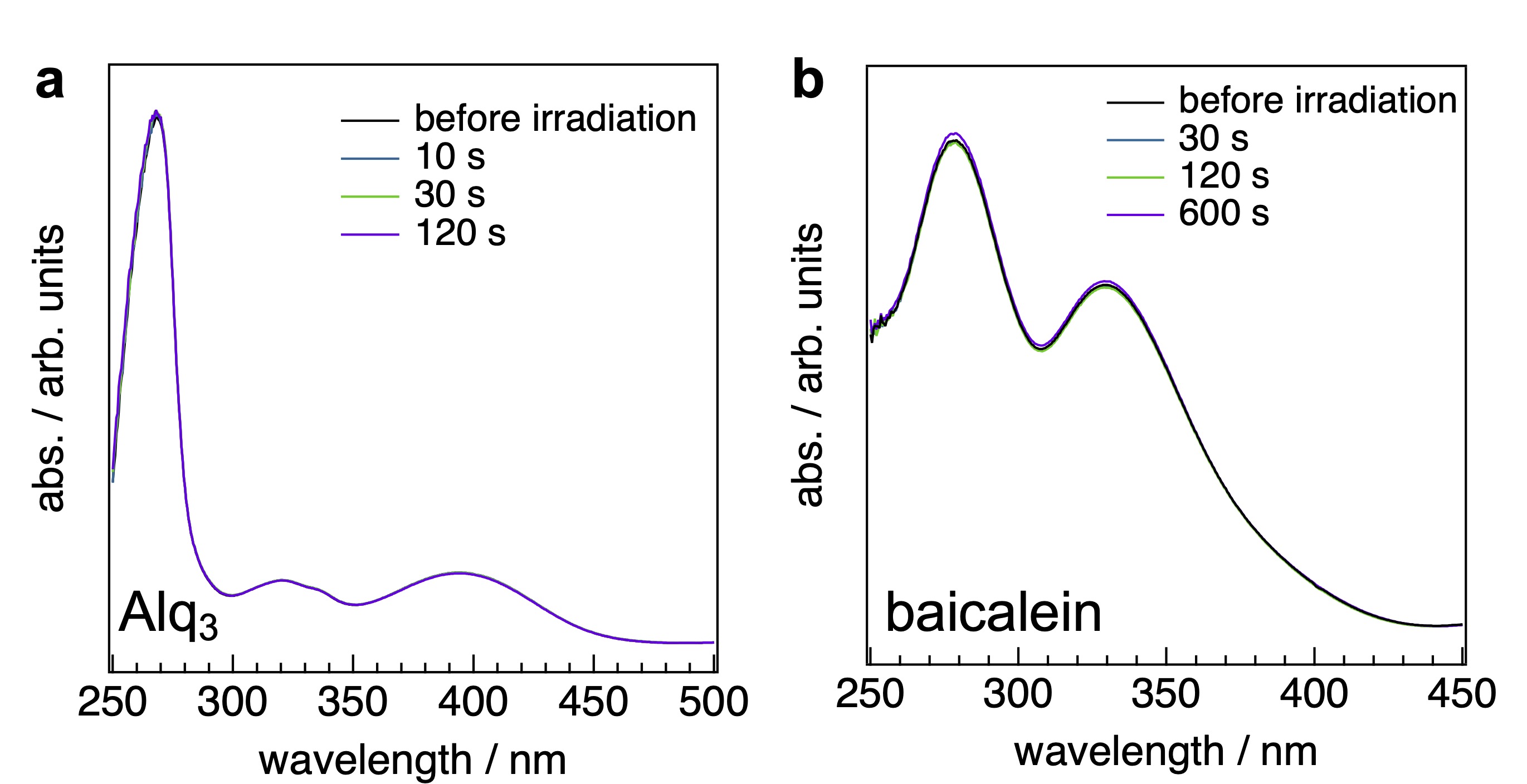


**Figure S2**. (a) and (b) show the evolution of UV-Visible spectra for Alq_3_ and baicalein films of 100 nm under the illumination of the simulated solar light at 1 sun. Both of Alq_3_ and baicalein films are tolerant against the simulated solar light.


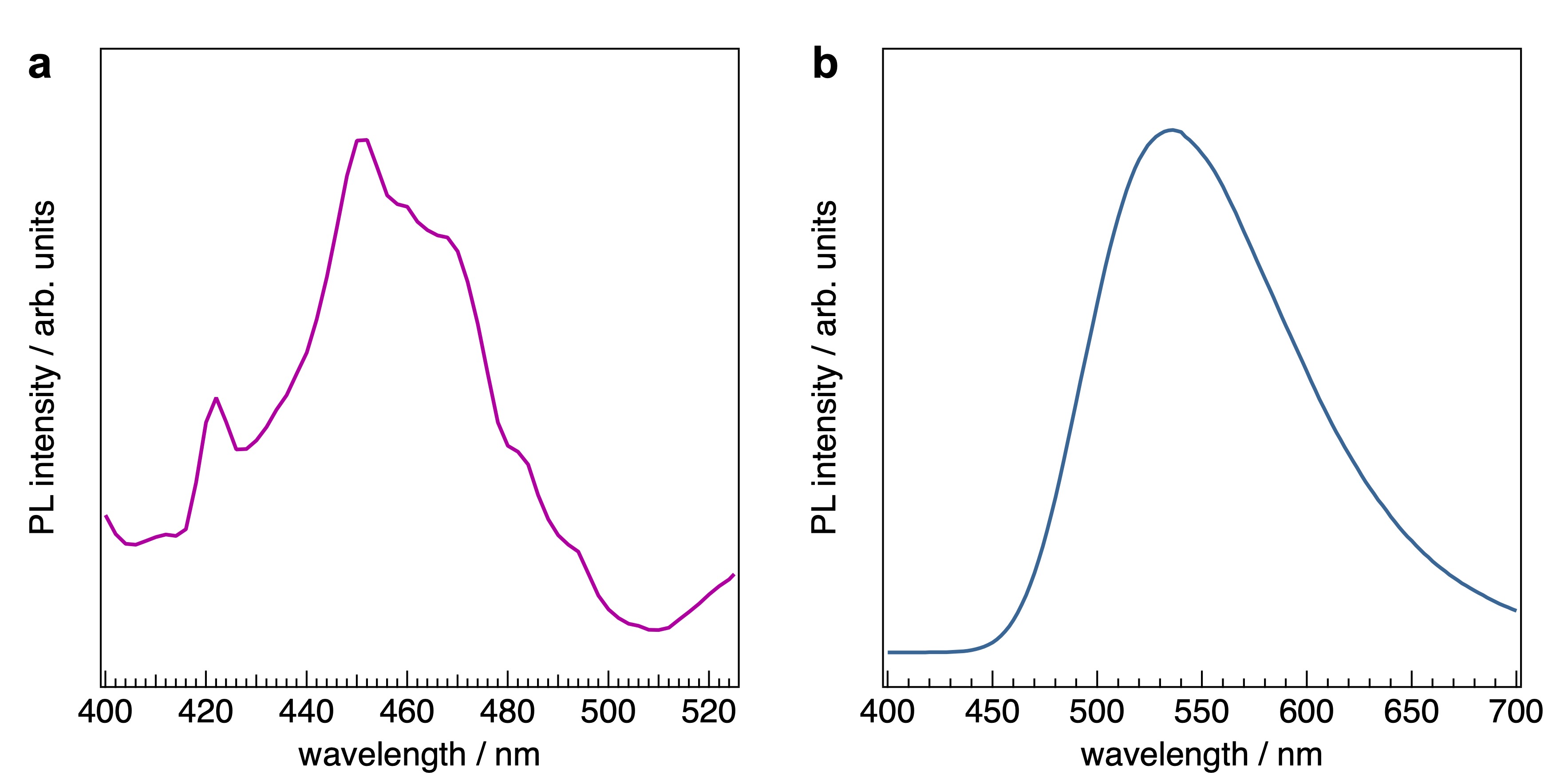


**Figure S3**. (a) and (b) show the steady-state PL spectra for baicalein and Alq_3_ evaporated films of 100 nm prepared on quartz substrates. The PL spectrum of the Alq_3_ film agrees well with the data previously reported^1^.

**Supplementary References**

1. M. Cölle and W. Brütting, *physica status solidi (a)*, **201**, 1095 (2004).
